# Influence of viral infection on the relationships between airway cytokines and lung function in asthmatic children

**DOI:** 10.1186/s12931-018-0922-9

**Published:** 2018-11-21

**Authors:** Toby C. Lewis, Ediri E. Metitiri, Graciela B. Mentz, Xiaodan Ren, Ashley R. Carpenter, Adam M. Goldsmith, Kyra E. Wicklund, Breanna N. Eder, Adam T. Comstock, Jeannette M. Ricci, Sean R. Brennan, Ginger L. Washington, Kendall B. Owens, Bhramar Mukherjee, Thomas G. Robins, Stuart A. Batterman, Marc B. Hershenson, the Community Action Against Asthma Steering Committee

**Affiliations:** 1 Departments of Pediatrics and Communicable Diseases, University of Michigan Medical School, 1150 W. Medical Center Dr., Building MSRB2, Room 3570B, Ann Arbor, MI 48109-5688 USA; 2 Molecular and Integrative Physiology, University of Michigan Medical School, Ann Arbor, USA; 3 Departments of Biostatistics, University of Michigan School of Public Health, University of Michigan, Ann Arbor, MI 48109 USA; 4 Environmental Health Sciences, University of Michigan School of Public Health, University of Michigan, Ann Arbor, MI 48109 USA; 5 Epidemiology, University of Michigan School of Public Health, University of Michigan, Ann Arbor, MI 48109 USA; 6 Health Behavior/Health Education, University of Michigan School of Public Health, University of Michigan, Ann Arbor, MI 48109 USA

**Keywords:** Asthma, Chemokine, Children, Cytokine, FEV_1_, FVC, Rhinovirus, Urban, Viral

## Abstract

**Background:**

Few longitudinal studies examine inflammation and lung function in asthma. We sought to determine the cytokines that reduce airflow, and the influence of respiratory viral infections on these relationships.

**Methods:**

Children underwent home collections of nasal lavage during scheduled surveillance periods and self-reported respiratory illnesses. We studied 53 children for one year, analyzing 392 surveillance samples and 203 samples from 85 respiratory illnesses. Generalized estimated equations were used to evaluate associations between nasal lavage biomarkers (7 mRNAs, 10 proteins), lung function and viral infection.

**Results:**

As anticipated, viral infection was associated with increased cytokines and reduced FVC and FEV_1_. However, we found frequent and strong interactions between biomarkers and virus on lung function. For example, in the absence of viral infection, CXCL10 mRNA, MDA5 mRNA, CXCL10, IL-4, IL-13, CCL4, CCL5, CCL20 and CCL24 were negatively associated with FVC. In contrast, during infection, the opposite relationship was frequently found, with IL-4, IL-13, CCL5, CCL20 and CCL24 levels associated with less severe reductions in both FVC and FEV_1_.

**Conclusions:**

In asthmatic children, airflow obstruction is driven by specific pro-inflammatory cytokines. In the absence of viral infection, higher cytokine levels are associated with decreasing lung function. However, with infection, there is a reversal in this relationship, with cytokine abundance associated with reduced lung function decline. While nasal samples may not reflect lower airway responses, these data suggest that some aspects of the inflammatory response may be protective against viral infection. This study may have ramifications for the treatment of viral-induced asthma exacerbations.

**Electronic supplementary material:**

The online version of this article (10.1186/s12931-018-0922-9) contains supplementary material, which is available to authorized users.

## Background

Geographic areas with high concentrations of low-income and minority ethnicity residents have high levels of asthma morbidity and mortality [1–3]. The factors leading to loss of asthma control and asthma exacerbations are complex. Previous studies have identified atopy, inadequate treatment, respiratory viral infections and environmental exposures as important drivers of asthma morbidity [4–7].

The effects of real-world respiratory viral infections on airway inflammation remain largely undefined. We [8] and others [9–12] have examined the innate immune response of children with asthma to natural colds. We found that nasal aspirate cytokine levels significantly increased in children with asthma. In addition, we found that a subset of cytokines (IFN-γ, CXCL8, CCL2, CCL4, CCL5, and CCL20) correlated with self-reported respiratory tract symptoms. However, we did not examine the influences of viral infection or nasal cytokines on airway function. Further, by limiting comparisons to samples taken during virus-negative well periods and viral-induced exacerbations, we ignored the potential effects of subacute viral infection. Finally, our cross-sectional study did not allow us to examine patterns of variables over time, which could provide more consistent information about the cytokines that drive lung function changes in chronic asthma.

The current study is drawn from an observational cohort of asthmatic school age children from Detroit who were concurrently enrolled in investigations examining the relationship between near-roadway exposures on asthma outcomes [13]. In addition to collecting measures of asthma health, we collected nasal lavage for detection of viral infection and measurement of respiratory tract cytokines and other biomarkers, and performed spirometry to assess lung function. We sought to determine the cytokines that drive reduced airflow, together with the influence of viral infections on these relationships. We hypothesized that children with greater respiratory tract inflammation would demonstrate worse airway function, and that viral infections would increase inflammation and negatively impact asthma symptoms and airway function. While our hypothesis was generally correct, we found that, in the presence of viral infection, higher levels of cytokines were associated with reduced lung function decline.

## Methods

### Study design and screening questionnaire

This study was conducted by Community Action Against Asthma (CAAA), a community-based participatory research (CBPR) partnership. School-age children with known or probable asthma were recruited using a screening questionnaire [14] distributed at community venues and through door-to-door recruitment in neighborhoods near highways. The questionnaire included demographic information, eight symptom questions, and if their child had ever been diagnosed by a medical care provider with any of the following conditions: asthma, bronchitis, bronchiolitis, reactive airways disease, pneumonia, or asthmatic bronchitis. In addition, parents were asked whether their child had taken prescription medication for any of these conditions in the last 12 months and, if so, whether they were taking these medications on a daily basis. Classification of asthma severity was based on symptom frequency and reported inhaled steroid use (Additional file 1: Table S1). This study was approved by the University of Michigan Medical School Institutional Review Board (IRBMED) (ID# HUM00018442) and conducted according to CBPR principles under the auspices of the CAAA Steering Committee.

### Data and sample collection

Fifty-three children participated in a two-week surveillance assessment period of health status each season from fall 2010 to summer 2011. During each two-week surveillance period, staff obtained spirometry, symptom reports and nasal lavage samples during three home visits. Respiratory symptoms were assessed using a modification of a previously developed respiratory symptom score [15] which assessed fever, cough, sore throat, nasal symptoms, wheezing, difficulty breathing and interference with usual activities (see Additional file 1: Table S2). Families were given a calendar and a simple respiratory symptom scale to mark the level of their symptoms.

From winter 2010 to summer 2011, measurements were repeated during a one-week period whenever the child experienced a symptomatic respiratory illness as defined by a symptom score of two or higher (referred to as a “sick period”). We intentionally set a low symptom threshold in order to maximize sensitivity to detect viral illnesses. Families contacted a central phone number when the child became ill. Staff would tally the symptom score over the phone and when symptoms reached the appropriate threshold, would begin a “sick period” assessment within 48 h of the phone call (median time to first sample was 72 h after the development of symptoms). Staff also conducted weekly telephone calls to identify illnesses in progress that families may not have called in to report, and would initiate a “sick period” collection if family reported that the child had current symptoms.

### Spirometry

Using protocols that we developed and successfully utilized in large-scale community-based asthma studies [7], staff conducted spirometry to assess lung function during home visits, using the EasyOne spirometer (NDD, Andover, MA). Additional details on spirometric procedures are described in Additional file 1.

### Nasal lavage

Nasal lavage samples were collected three times during a two-week surveillance period or three times during a one-week sick period by the field staff. Two squirts of isotonic 0.65% sodium chloride (estimated to be < 1 ml per nostril, B.F. Ascher, Lenexa, KS) were instilled into the child’s nostrils Subjects then blew their nose into a zippered plastic bag, and three ml of M4RT viral transport medium (Remel, Lenexa, KS) was added. After collection, samples were double bagged, placed in a transport cooler at 0 °C, conveyed to a local laboratory (Henry Ford Health System Epidemiology Lab) for preliminary processing and freezing to − 70 °C, and subsequently transported to Ann Arbor on dry ice.

### Detection of respiratory viruses

Whole nasal lavage samples were homogenized using a hand held homogenizer (Thermo Fisher Scientific, Waltham, MA). Nucleic acids were extracted using TRIzol-LS (ThermoFisher, Waltham, MA), chloroform and an RNeasy Mini Kit (Qiagen, Valencia, CA). Samples were analyzed for viral nucleic acid by multiplex PCR using the Seeplex RV-15 ACE detection kit (Seegene, Concord, CA). This kit detects human adenovirus, bocavirus 1–4, coronaviruses 229E/NL63 and OC43, enterovirus, influenza A and B, metapneumovirus, parainfluenza viruses 1–4, RSV A and B and rhinovirus A, B and C. For surveillance samples, all specimens were analyzed for virus; for sick samples, specimens from the same sick week were pooled prior to analysis (samples from sick periods were not pooled for cytokine or viral copy number determination, see below).

### Nasal lavage mRNA and protein expression

All nasal lavage samples were analyzed for mRNA and protein. cDNA was synthesized from total RNA by Taqman reverse transcriptase kit (Qiagen). DNA was digested with DNase I (Qiagen). CXCL8, CXCL10, IRF7, RIG-I, MDA5, TLR3 and IFN-λ1 mRNA expression were measured by qPCR. Specific primers and probes spanning exon-exon junctions (intron splice-sites) were used to prevent amplification of genomic DNA (Additional file 1: Table S3, Online Repository). Expression levels were normalized to GAPDH using the ΔΔCt method. Reactions with a cycle number higher than 35 were not included in the analysis. CXCL8, CXCL10, CCL2, CCL4, CCL5, CCL20, CCL24, IL-4, IL-13 and soluble ICAM-1 (sICAM) protein levels were determined by multiplex immune assay (Affymetrix, Santa Clara, CA). Minimum detection levels for the proteins assayed are provided in the Additional file 1: Table S4. Biomarkers were chosen based on previous studies showing elevations after RV infection, our interest in examining biomarkers we had not previously studied, difficulty of detecting some biomarkers or cytokines in the nasal aspirate fluid, cost and availability.

### Statistical analysis

Assessment of the distribution of spirometric data and nasal lavage biomarkers was conducted using means, medians, histograms and QQ plots (data not shown). Because of strong right skewedness, biomarker values were natural log transformed for subsequent analyses. The effects of viral infection on group median nasal lavage mRNA and protein expression were analyzed by the Wilcoxon Median Test. We chose to use a non-parametric test because the transformed cytokine distributions were still slightly skewed, with a number of zero values and high outliers which we did not exclude from the analysis. In addition, because we divided subjects and samples by asthma severity, we had a small sample size for some measures. Finally, in the case of TLR3 and IFN-λ1 mRNAs, which were detected in only 35 and 11% of the samples, data were analyzed as binary variables by Fisher’s exact test.

For our primary analyses, we evaluated the strength of association between mean respiratory tract inflammatory responses and pulmonary function using generalized estimating equations (GEE) with an exchangeable correlation structure using the identity link in the case of continuous outcomes and the log link for binary ones (SAS, Cary, NC). This design allowed us to test the influence of cytokine on lung function with and without virus. GEE was used rather than logistic or Poisson models because of the longitudinal nature of the data and the repeated observations for each child [16, 17]. The GEE procedure can be used for small populations [18]. All individual samples (virus-negative and virus-positive “surveillance samples,” virus-negative and virus-positive “sick samples”) were included in the analysis. We adjusted for covariates including age, gender, ethnicity/race, smoker in the home, caregiver educational attainment, self-reported atopy, caregiver depressive symptom score, season and whether the sample was from a surveillance or sick period. Family income, baseline asthma severity, medication use and proximity to high-traffic highways were evaluated but not included in final models as they were not significant predictors or were collinear with other covariates in the model. In a secondary analysis, linear plots of lung function and cytokine level when virus was either absent or present were generated. Additional details on statistical analysis are described in Additional file 1.

## Results

### Study participants

Fifty-three children between 5 and 12 years-old were enrolled. Subjects predominantly self-identified as African-American (Table 1). The majority was reported by their parent/caregiver to be atopic, exposed to tobacco smoke and live in a household with an income ≤ $15,000. Most children reported symptoms or medication use consistent with mild intermittent or mild persistent asthma, but one-quarter had moderate-to-severe persistent disease. At enrollment, one-quarter of the subjects were taking inhaled corticosteroids or used them within the last year. Group mean FVC, FEV_1_ and PEF during the first surveillance period were in the range of normal, but FEV_1_/FVC ratio and FEF_25–75_ were reduced (Table 2). It should be noted that, although all children met initial criteria for participation, only 47 of 53 children performed acceptable expiratory maneuvers for spirometric analysis.

**Table 1:** Participant baseline characteristics (*n* = 53)

Age in years, mean (SD)	9.7 (2.1)
Female gender, n (%)	23 (43.4)
Race, Non-Hispanic African-American, n (%)	46 (86.8)
Household income ≤ $15,000, n (%)	30 (56.6)
Caregiver years of education ≤12, n (%)	30 (56.6)
Caregiver depression CESD score, mean (SD)	8.8 (5.1)
Smoker in household, n (%)	36 (67.9)
Asthma severity, N (%)	
Moderate or severe persistent	14 (26.4)
Mild persistent	27 (50.9)
Mild intermittent	12 (22.6)
Atopy (self-reported), yes (%)	38 (71.7)
Any asthma medication use in last 12 months, n (%)	
Inhaled corticosteroids	12 (22.6)
Short acting bronchodilator only	21 (39.6)
No asthma medication	20 (37.7)
Asthma control test (ACT) score, mean (SD)	20.0 (4.2)

**Table 2:** Baseline surveillance valid health measures

	N	Median (Range)
Symptom Score	53	2.3 (0, 27)
Lung function (% of predicted)	N	Mean (SD)
FVC	43	98.5 (17.0)
FEV_1_	43	90.3 (18.3)
FEV_1_/FVC ratio	43	79.3 (6.6)
FEF_25–75_	42	70.8 (21.9)
PEF	42	91.3 (19.1)

### Participant respiratory illnesses

From September 2010 to August 2011, 392 surveillance samples were collected, 105 (26%) of which were positive for one or more viruses. From December 2010 to August 2011, there were 85 self-reported respiratory illnesses, for which 233 samples were collected. Analysis of pooled samples for each illness showed that 30 (35%) of these illnesses were positive for at least one virus. RV was detected in 54 out of 105 (51%) of the virus-positive surveillance samples and 23 out of 30 (77%) of virus-positive sick illnesses (Table 3).

**Table 3:** Participant viral infections

	N	% of total
Surveillance collection (*N* = 410)		
No virus	288	70.2
Virus	94	22.9
Rhinovirus		
Single infections	46	11.2
Multiple infections	4	1.0
Non-rhinovirus		
Single infections^a^	39	9.5
Multiple infections	5	1.2
Cold collection (number of pooled samples = 92)		
No virus	55	60.0
Virus	28	30.4
Rhinovirus		
Single infections	20	21.7
Multiple infections	2	2.2
Non-rhinovirus		
Single infections^b^	6	6.5
Multiple infections	0	0.0

^a^Coronavirus 229E/NL63 (9), RSV A (8), coronavirus OC43 (5), RSV B (4), influenza A (4), influenza B (3), adenovirus (2), metapneumovirus (2), parainfluenza 2 (2)

^b^Influenza A (2), influenza B, coronavirus 229E/NL63, parainfluenza 2, RSV B

### Effects of viral infection on lung function

**Fig. 1:**
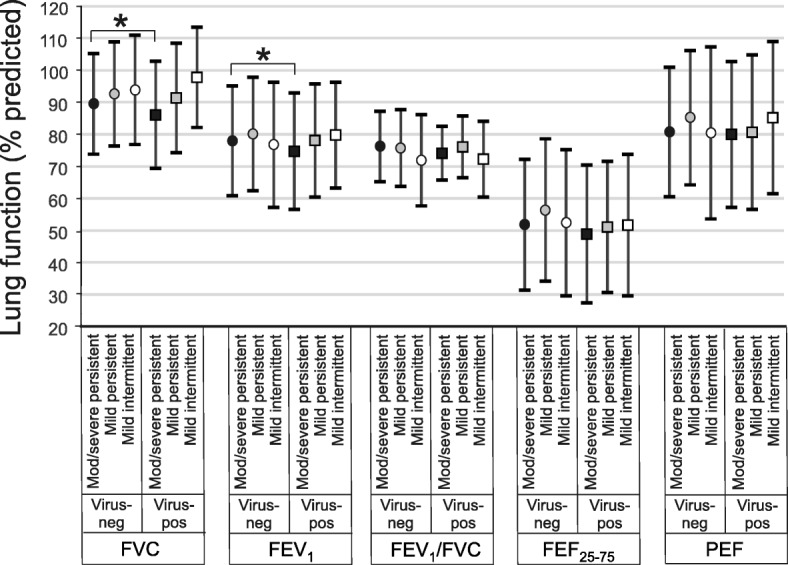
Effect of viral infection on spirometry in subjects with moderate-to-severe persistent (black symbols), mild persistent (grey symbols) and mild intermittent asthma (white symbols). FVC, FEV1, FEF25–75 and PEF are shown (mean ± SD). GEE models were used for pairwise comparison of the means (*p < 0.05)

We examined the influence of asthma severity on the lung function response to viral infection. When we divided subjects into mild intermittent, mild persistent and moderate-to-severe persistent asthma, we found that subjects with moderate-to-severe persistent asthma experienced significant (*p* < 0.05) reductions in FVC and FEV_1_ after viral infection (Fig. 1). Changes in FEV_1_/FVC (*p* = 0.08) and FEF_25–75_ (*p* = 0.09) did not reach statistical significance.

### Effects of viral infection on nasal lavage biomarkers

All nasal samples were analyzed for mRNA expression of CXCL8, CXCL10, IFN-λ1, TLR3, MDA5, RIG-I and IRF7. Samples were also analyzed for CXCL8, CXCL10, IL-4, IL-13, sICAM-1, CCL2, CCL4, CCL5, CCL20 and CCL24 protein. The median individual surveillance and sick period samples analyzed per subject was 7 (range, 1–12) and 3 (range, 2–15), respectively. MDA-5, RIG-I, IRF7, CXCL10 and CXCL8 mRNA were detected in the 73–99% of the samples, whereas TLR3 and IFN-λ1 mRNA were detected in 35 and 11% of the samples, respectively.

The effect of viral infection on group median cytokine data are shown in Table 4. Viral infections were associated with significantly increased mRNA expression of CXCL10, RIG-I and MDA5 and protein expression of all biomarkers tested. In addition, viral infections were associated with an increase in the percentage of samples with detectable TLR3 and IFN-λ1 mRNA. Responses to RV and non-RV infections were generally similar, except for TLR3 mRNA and CXCL8 protein, which were not increased in non-RV infections (data not shown).

**Table 4:** Effect of viral infection on nasal lavage mRNA and protein expression

	Virus-negative			Virus-positive			
mRNA	N	Median	IQR	N	Median	IQR	*p*-value
CXCL8	404	4.68	(2.15, 9.71)	174	5.01	(2.45, 10.78)	0.54
CXCL10	404	0.001	(0.00, 0.01)	174	0.0098	(0.001, 0.06)	< 0.01
IRF7	404	0.06	(0.02, 0.14)	174	0.06	(0.02, 0.14)	0.99
RIG-I	404	0.01	(0.00, 0.03)	174	0.02	(0.00, 0.05)	0.04
MDA5	404	0.01	(0.00, 0.03)	174	0.02	(0.01, 0.07)	< 0.01
Binary	N	Detected (%)		N	Detected (%)		p-value
TLR3	404	132/404 (32.7)		174	69 (39.7)		0.11
IFN-λ1	404	30/404 (7.4)		174	33 (19.0)		< 0.01
Protein	N	Median	IQR	N	Median	IQR	p-value
CXCL8	433	177.00	(71.5, 431.6)	172	253.35	(97.25, 868.9)	< 0.01
CXCL10	429	397.90	(213.9, 656.2)	171	703.20	(419.5, 1553.5)	< 0.01
IL-4	436	15.60	(4.9, 51.65)	175	31.10	(9.3, 75.2)	< 0.01
IL-13	428	4.60	(0.0, 27.1)	167	18.80	(0.0, 37.8)	< 0.01
sICAM1	431	293.20	(100.8, 643.6)	170	560.20	(207.6, 1219.2)	< 0.01
CCL2	433	71.40	(26.9, 132.9)	170	103.70	(40.3, 219.6)	< 0.01
CCL4	426	300.9	(40.3, 1261.9)	170	1198.65	(163.0, 3050.6)	< 0.01
CCL5	433	5.00	(0.0, 18.1)	174	9.55	(0.0, 27.4)	< 0.01
CCL20	439	290.3	(78.0, 651.1)	178	580.0	(186.1, 1184.5)	< 0.01
CCL24	434	4.47	(1.06, 14.24)	175	9.16	(2.56, 21.06)	< 0.01

Levels of mRNA expression are normalized to GAPDH. Group median data are shown except for TLR3 and IFN-λ1 mRNA, for which results were analyzed as binary variables. Samples were divided into virus-negative and virus positive. Differences were analyzed by the Wilcoxon Median Test except for TLR3 and IFN-λ1 which were analyzed by Fisher’s exact test

**Fig. 2:**
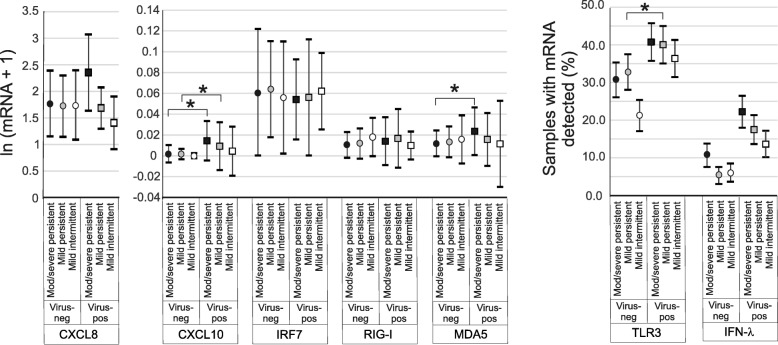
Effect of viral infection on nasal lavage mRNAs by asthma severity. mRNA expression was measured by qPCR and normalized by GAPDH. Medians ±IQR are shown. TLR3 and IFN-λ1 mRNA results were analyzed as binary variables (proportions and 95% confidence intervals are shown. Pairwise comparisons of medians were performed using the Wilcoxon Rank-Sum Test (red squares, moderate-to-severe persistent asthma; blue squares, mild persistent asthma; green squares, mild intermittent asthma; *p < 0.05, †0.05 < *p* < 0.10)

Next, we examined the influence of asthma severity on the cytokine responses to viral infection. In subjects with moderate-to-severe persistent asthma, viral infection was associated with significant increases in mRNA expression of CXCL10 and MDA5 (Fig. 2). Viral infection was also associated with increased mRNA expression of CXCL10 in subjects with mild persistent asthma. Viral infection was associated with an increase in the number of samples with detectable TLR3 mRNA expression in subjects with mild persistent asthma. Finally, compared to other virus-positive groups, the number of IFN-λ1 mRNA-positive samples tended to be higher in infected subjects with moderate-to-severe asthma (*p* = 0.09).

**Fig. 3:**
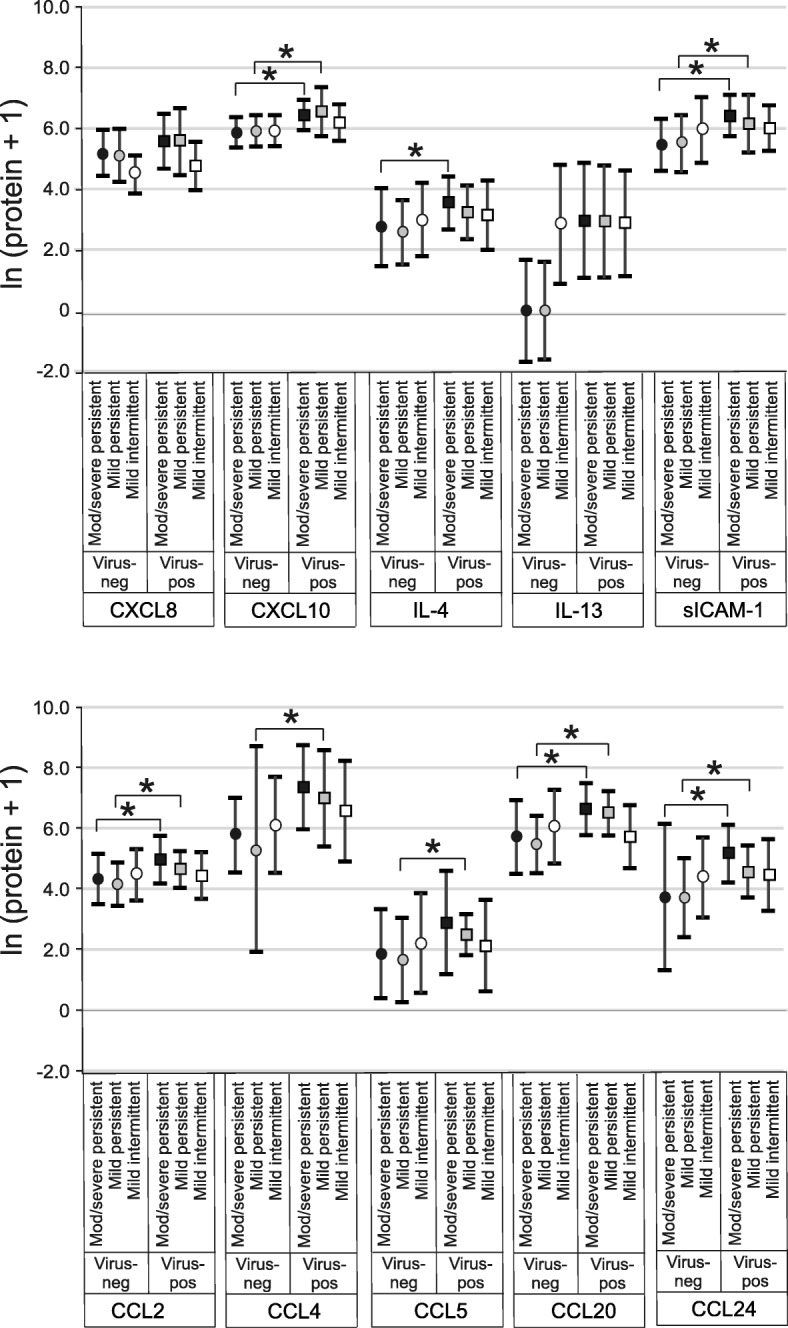
Effects of viral infection on nasal lavage cytokine concentrations by asthma severity (median ± IQR). Cytokines were measured by multiplex immune assay. Pairwise comparisons of medians were performed using the Wilcoxon Rank-Sum Test (black symbols, moderate-to-severe persistent asthma; grey symbols, mild persistent asthma; white symbols, mild intermittent asthma; *p < 0.05)

In moderate-to-severe persistent asthmatics, viral infection was associated with increased protein abundance of CXCL10, IL-4, sICAM-1, CCL2, CCL20 and CCL24 (Fig. 3). In mild persistent asthmatics, viral infection was associated with increased protein abundance of CXCL10, sICAM-1, CCL2, CCL4, CCL5, CCL20 and CCL24. Virus-positive samples from subjects with mild intermittent asthma showed no significant increases in protein expression.

### Associations of nasal lavage biomarker, viral infection and lung function

Next, we evaluated the strength of association between respiratory tract inflammatory responses and pulmonary function using GEE, including an interaction term between viral presence and inflammatory marker. Most unexpectedly, we found frequent and strong interactions between biomarkers and virus on lung function. For FVC, statistically significant interactions between viral infection and inflammation were seen for CXCL10 mRNA, TLR3 mRNA, IL-4, IL-13, CCL5, CCL20 and CCL24 (Additional file 1: Table S5). The interaction between viral infection and CCL4 approached statistical significance. In contrast, CXCL10 protein showed a negative association with FVC independently of infection.

**Fig. 4:**
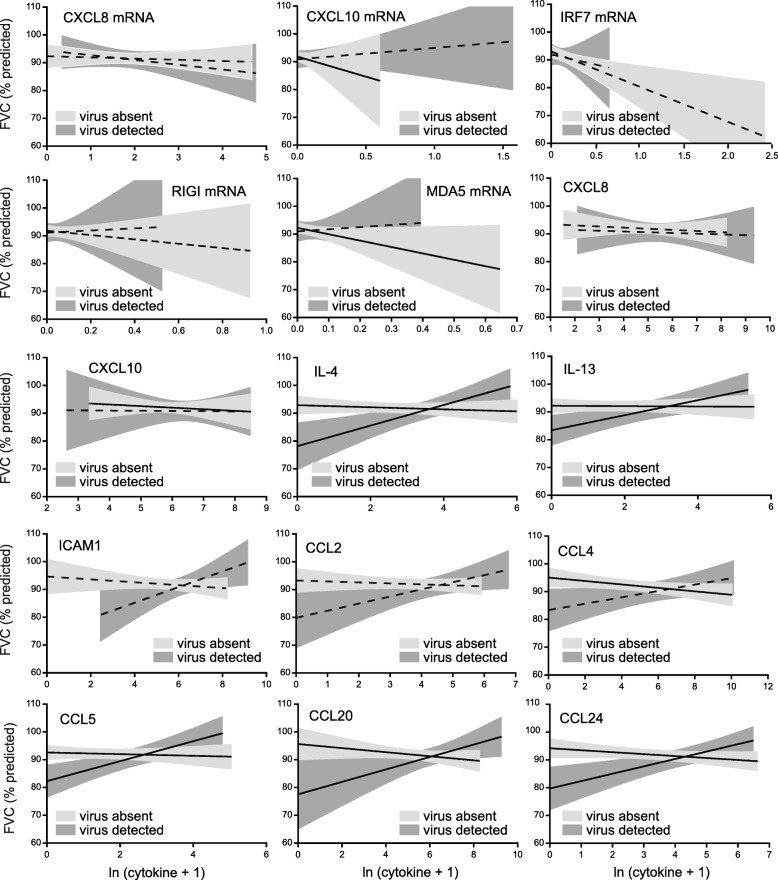
Effect of viral infection on the relationships between log-transformed nasal lavage biomarker levels and percent predicted FVC. In the absence of virus, we found negative associations between biomarker level and FVC (unadjusted 95% confidence intervals are shown in light grey, solid lines indicate a statistically significant association between cytokine and FVC by GEE; dashed lines indicate no statistically significant association). However, in the presence of virus, increasing levels of biomarker had a positive effect on FVC (unadjusted 95% confidence intervals are shown in dark grey; solid lines indicate a statistically significant association). For clarity individual data points are not shown here, but may be found in Additional file 2: Figure S1

Figure 4 shows the graphical relationships between nasal lavage biomarker levels and FVC in the absence and presence of viral infection. (Panels for the binary variables TLR3 and IFN-λ mRNA are not shown.) In the absence of virus, there were significant negative associations between biomarker and FVC for CXCL10 mRNA, MDA5 mRNA, CXCL10, IL-4, IL-13, CCL4, CCL5, CCL20 and CCL24. In the presence of virus, there were significant positive associations between biomarker and FVC for IL-4, IL-13, CCL5, CCL20 and CCL24.

**Fig. 5:**
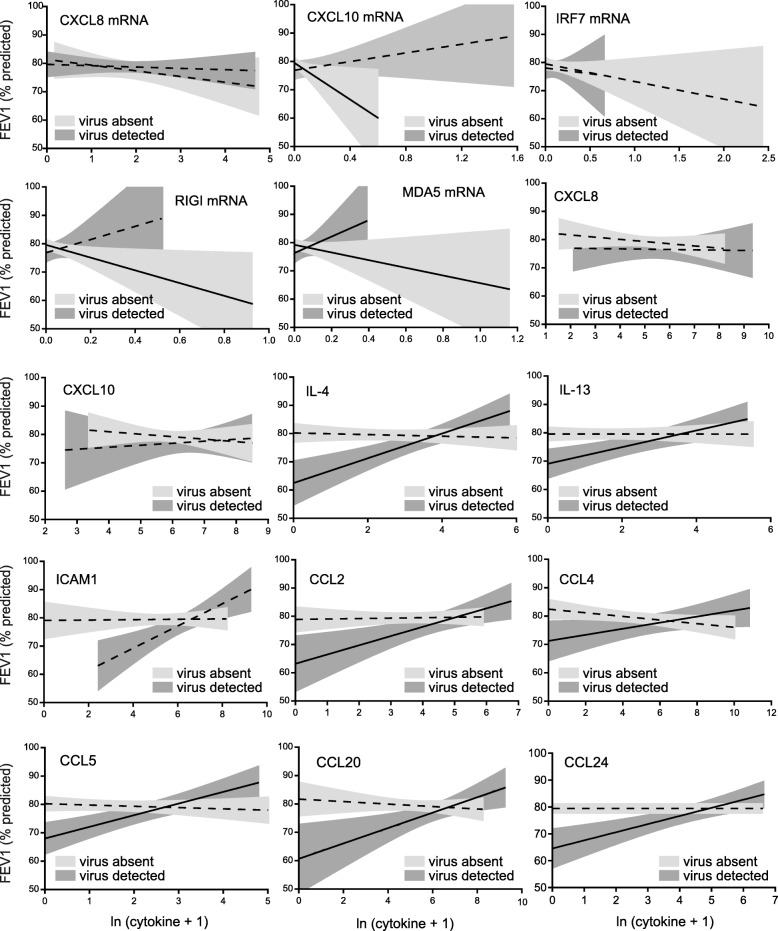
Effect of viral infection on the relationships between log-transformed nasal lavage biomarker levels and percent predicted FEV1. In the absence of virus, we found negative associations between biomarker level and FEV1 (95% confidence intervals are shown in light grey, with solid lines indicating a significant association between cytokine and FEV1 by GEE; dashed lines indicate no statistically significant association). In the presence of virus, increasing levels of biomarker had a positive effect on FEV1 (95% confidence intervals are shown in dark grey; solid lines indicate a statistically significant association). For clarity individual data points are not shown here, but may be found in Additional file 3: Figure S2

Longitudinal analysis also showed a significant influence of viral infection on the associations between biomarkers and FEV_1_ (Additional file 1: Table S6). For FEV_1_, statistically significant interactions between viral infection and inflammation were seen for CXCL10 and TLR3 mRNA, IL-4 and CCL24 levels. Interactions with CCL4 and CCL5 approached statistsical significance. CXCL10 protein levels had negative associations with FEV_1_, independent of viral infection. Again, these patterns are discerned graphically (Fig. 5). In the absence of virus, there were significant negative associations between biomarker and FEV_1_ for CXCL10, RIG-I and MDA5 mRNA. In the presence of virus, there were significant positive associations between biomarker and FEV_1_ for MDA5 mRNA, IL-4, IL-13, sICAM-1, CCL2, CCL5, CCL20 and CCL24.

The influence of viral infection on the relationships between nasal lavage biomarkers and FEV_1_/FVC, FEF_25–75_ and PEF are fully described in this article’s Online Repository (Additional file 1: Tables S7-S9). For these lung function parameters there was less of an effect of viral infection on cytokine level. For FEV_1_/FVC, FEF_25–75_ and PEF, statistically significant interactions between viral infection and inflammation were seen for CXCL10 mRNA. For PEF, interactions between viral infection and RIG-I and TLR3 mRNA were also seen. RIG-I mRNA had a negative association with FEV_1_/FVC independent of viral infection. CXCL8 mRNA and protein levels had a negative association with FEF_25–75_ independent of viral infection. RIG-I and MDA5 mRNA had negative associations with PEF independent of viral infection. Finally, viral infection was strongly associated with significant reductions in FEF_25–75_.

## Discussion

Previous studies have shown increased abundance of airway cytokines following both natural [8–12, 19, 20] and experimental [21, 22] colds. In this report, we examine the interactive effects of viral infection and airway cytokines on lung function in patients with asthma. We anticipated that, in the absence of virus, children with greater airway inflammation would demonstrate worse airway function. Consistent with this, in the absence of viral infection, several nasal lavage biomarkers correlated negatively with lung function. For example, CXCL10 mRNA, MDA5 mRNA, CXCL10, IL-4, IL-13, CCL4, CCL5, CCL20 and CCL24 were each negatively associated with FVC. We also expected that viral infections would increase inflammation and negatively impact airway function. Accordingly, we found that viral infection had independent positive associations with nearly all nasal lavage biomarkers and, in subjects with moderate-to-severe persistent asthma, negative influences on FVC and FEV_1_. However, we found many cases in which there was a significant interactive effect between viral infection and lung function, in that the simultaneous presence of viral infection and high cytokine levels was associated with less severe reductions in lung function. Among other associations, in the presence of a virus, IL-4, IL-13, CCL5, CCL20 and CCL24 each positively correlated with both FVC and FEV_1_. Thus, whereas airway inflammation is associated with reduced lung function in the absence of viral infection, cytokine expression is associated with a diminution of lung function deficits caused by viral infection.

Cytokines promote proliferation and chemotaxis of phagocytes and granulocytes expressing antiviral substances including granule proteins, antimicrobial peptides, proteolytic enzymes and reactive oxygen species, consistent with a protective function. On the other hand, an exuberant response to RV, a virus that infects relatively few airway cells [23, 24] and possesses minimal cytotoxic effects compared to influenza or adenovirus [25], could be maladaptive. In a mouse model of RV infection, CXCR2 [26] and TLR2 [27] null mice both show reduced airway inflammation and responsiveness but no change in viral load, suggesting that cytokines mediate RV-induced tissue injury. However, in the present study, we show that, in the context of respiratory viral infection, higher expression of pro-inflammatory cytokines is associated with less severe reductions in lung function. This study may have ramifications for the treatment of viral-induced asthma exacerbations. For example, treatments aimed at reducing the expression of specific pro-inflammatory cytokines may reduce symptoms but also attenuate the potential protective effects of these cytokines on lung function. Indeed, a recent multicenter study of children admitted to the emergency department for asthma exacerabtions showed that viral detection was associated with failure of management with oral corticosteroids and inhaled bronchodilators [28].

As noted above, we also obtained important information regarding the influence of airway cytokines on lung function in the absence of viral infection. IL-4, IL-13 and other type 2 cytokines have long been known to be drivers of allergic asthma [29, 30], and many studies have shown increased chemokine levels in the airways of patients with asthma compared to controls [31–39]. In our longitudinal study, we found that, in the absence of viral infection, CXCL10, IL-4, IL-13, CCL4, CCL5, CCL20 and CCL24 were each negatively associated with FVC. We also found that CXCL10 mRNA MDA5 mRNA and IFN-λ1 mRNA were each negatively associated with FVC and FEV_1_. mRNA expression of CXCL10 and MDA5 are each known to be induced by IFN-γ, a type 1 cytokine [40, 41]. While we did not measure IFN-γ, these data suggest a potential negative influence of this cytokine on lung function in urban children with chronic asthma in the uninfected state. Levels of bronchoalveolar IFN-γ, a canonical type 1 cytokine, were recently associated with severe asthma [42]. Exhaled breath IFN-γ was noted to be a significant indicator of childhood asthma severity, as measured by FEV_1_ [43].

We were initially surprised that airway cytokines correlated with FVC more strongly than pulmonary function parameters. However, FVC has previously been noted to negatively correlate with outdoor and indoor environmental exposures in children [44–47] and adults [48]. FVC in children has also been negatively correlated with various biomarkers including airway macrophage carbon content [45], exhaled breath malondialdehyde [46] and sputum dipalmitoylphosphatidylcholine [49].

We also obtained new information on the influence of asthma severity on the response to viral infection. Virus-induced induction of airway cytokines was not decreased in children with moderate-to-severe persistent asthma compared to subjects less severe disease, and in many cases viral-induced increases in cytokine level were significant in subjects with persistent asthma but not subjects with intermittent asthma. While there were significant overlapping ranges between the asthma severity groups, these data, obtained from asthmatic subjects experiencing natural colds, do not support the concept that patients with severe asthma have deficient antiviral responses [50–52]. On the other hand, our data are consistent with a recent study showing that, following experimental RV infection, subjects with allergic asthma show greater levels of nasal and bronchial cytokines, chemokines and IFNs compared to control subjects [53]. In addition, the absence of a reduction in viral-induced mRNA or protein expression in children with moderate-to-severe persistent asthma demonstrates that the positive associations we found between nasal lavage cytokine level and lung function are not due to increased cytokine production in subjects with better lung function.

There are several limitations to our study. First, we used nasal lavage to sample the respiratory tract of children with asthma. This method allowed repeated collection of samples from children as young as 5 years-old in a relatively non-invasive manner. While previous studies have shown that gene expression among asthmatic children is altered similarly in nasal and bronchial airways [53, 54], measurements of nasal lavage cannot actually represent the airway biology, and we did not validate our method by comparing our results with lower respiratory tract specimens such as sputum, breath condensate or bronchoalveolar lavage. Second, we studied a relatively small number of children (*n* = 53). Third, we found that 26.1% of surveillance samples were positive for virus, compared to 33.7% of self-reported respiratory illnesses. Thus, subjects were only slightly more likely to have a virus during self-reported sick periods than during surveillance sample collection. We believe the low viral detection rate during symptomatic episodes can be explained by the fact that samples from symptomatic illnesses were not collected in the fall and instead were only collected from January to August, when rhinovirus infections are less prevalent. Cold-like illnesses were unlikely to represent false-negative viral infections, as they were unaccompanied by increases in nasal aspirate MDA5, a double-stranded RNA pattern recognition receptor which was increased in virus-positive samples and has been shown to be induced following RV infection [55]. Nor were cold-like illnesses associated with reduced pulmonary function. Also, the symptom threshold for “sick” sample collection was quite low (2 or more). The reasoning behind this was to increase the likelihood of capturing viral illnesses for study. We also offered a $50 financial reimbursement for each sick period assessment which served to offset the family time and effort needed to participate in sick assessment/nasal secretion collection. These design elements contributed to a low viral detection rate. For example, a symptom score of two could be achieved simply with a runny and stuffy nose, which could easily represent allergic rhinitis rather than a cold. Fourth, despite extensive training and coaching in spirometry, many expiratory maneuvers were excluded due to inadequate data quality. We have evaluated the impact of our data cleaning procedures and found no systematic exclusion of children compared to the overall study population, and thus feel these procedures are unlikely to introduce selection bias. Fifth, the current analysis evaluates associations between biomarker levels, viral detection, and spirometry obtained on the same day, and does not evaluate for the possibility of lagged effects. Sixth, some of our assessments, such as medication use, asthma severity, and atopy, were based on self-report and were not independently validated, leaving open the possibility of measurement error. Seventh, our data from urban children with asthma may not be generalizable to other groups of asthmatic children. As we have shown previously [14], these children tend to be undertreated, as reflected by the number of subjects who were not taking medication (and therefore labeled as “intermittent asthmatics”). Urban children with asthma who are not treated with inhaled steroids may be more susceptible to viral-induced reductions in airway function than suburban children [56, 57]. Eighth, we did not take into account bacterial colonization in our study. Bacterial colonization could alter the response to rhinovirus infection [58].

Finally, while we found that proximity to high-traffic highways was not a significant predictor of lung function or the relationship between viral exposure and lung function, we have not yet formally examined the effect of specific exposures. Detroit has a history of elevated air pollution and nonattainment of the ozone and particulate matter standards [59]. We previously examined relationships between lung function and ambient levels of ozone and particulate matter in a longitudinal cohort study of school-age children with asthma in Detroit [7]. In a two-pollutant model including PM_10_ and O_3_, there was an association between O_3_ exposure and diurnal variation of FEV_1_ 1 day after exposure in children without self-reported cold symptoms and a larger odds ratio for children with cold symptoms. It is therefore conceivable that specific pollutants could have intensified the effects of viral infection on lung function.

## Conclusions

We conclude that in urban children with asthma, in the absence of respiratory virus, selected nasal lavage cytokines are significantly associated with reduced lung function. These data firmly establish the link between airway inflammation and asthma severity in a longitudinal cohort of children with stable asthma. However, in the presence of viral infection, there is a reversal in the relationship between nasal cytokines and clinical outcome, with higher levels correlating with less severe reductions in lung function. While nasal samples may not reflect lower airway responses, these data suggest that some aspects of the inflammatory response may be protective against viral infection. This study provides new insight into the host response to respiratory viral infections, and may have ramifications for the treatment of viral-induced asthma exacerbations.

## Additional files

Additional file 1: Supplemental text and tables. (DOCX 71 kb)

Additional file 2: Figure S1. (PDF 13647 kb)

Additional file 3: Figure S2. (PDF 14140 kb)
