# Supplementary material for: Influence of viral infection on the relationships between airway cytokines and lung function in asthmatic children
Source: Respir Res. 2018 Nov 21;19:228. doi: 10.1186/s12931-018-0922-9 (PMC6249926; doi:10.1186/s12931-018-0922-9)
Supplement: Supplementary file 1 — Supplemental text and tables. (DOCX 71 kb) [file 12931_2018_922_MOESM1_ESM.docx]

Additional file 1

**Influence of viral infection on the relationships between airway cytokines and lung function in asthmatic children**

Toby C. Lewis, Ediri E. Metitiri, Graciela B. Mentz, Xiaodan Ren, Ashley R. Carpenter, Adam M. Goldsmith, Kyra E. Wicklund, Breanna N. Eder, Adam T. Comstock, Jeannette M. Ricci, Sean R. Brennan, Ginger L. Washington, Kendall B. Owens, Bhramar Mukherjee, Thomas G. Robins, Stuart A. Batterman, Marc B. Hershenson and the Community Action Against Asthma Steering Committee**Methods**

*Spirometry*. Using protocols that we developed and successfully utilized in large-scale community-based asthma studies morbidity [[1](#_ENREF_1)], staff conducted spirometry to assess lung function during home visits, using the EasyOne spirometer (NDD, Andover, MA). Prior to enrollment in the longitudinal study, eligible children participated in a two-week “wash-in” assessment period, during which quality and quantity of exhalation maneuvers was assessed. Only children meeting standardized criteria continued participating in the longitudinal portion of the study. At the beginning of each data collection period, a technician observed the child’s technique and provided reinforcement training. An extensive data cleaning process was conducted in which each flow-volume loop was assessed by a trained technician for quality, and each session scored for acceptability and reproducibility of at least two exhalations. A subset of curves was assessed by more than one evaluator to assess inter-rater reliability and to improve quality control. Data of insufficient quality or quantity were excluded. Forced vital capacity (FVC), forced expiratory volume in 1 second (FEV_1_), forced expiratory flow at 25-75% of exhaled volume (FEF_25-75_) and peak expiratory flow (PEF) were assessed and expressed as percent predicted for height using NHANES prediction equations [[2](#_ENREF_2)]. Extreme values were excluded (defined as greater than 150% predicted or lower than 30% predicted for FVC, FEV_1_, and PEF, greater than 145% predicted and less than 15% predicted for FEF_25-75_, and greater than 100% for FEV_1_/FVC ratio). EasyOne spirometers meet ATS diagnostic standards, and have been shown to have excellent accuracy, precision, and stability of readings over time [[3](#_ENREF_3)].

*Statistical analysis*. Assessment of the distribution of spirometric data and nasal lavage biomarkers was conducted using means, medians, histograms and QQ plots (data not shown). Because of strong right skewedness, biomarker values were natural log transformed for subsequent analyses. The effects of viral infection on group median nasal lavage mRNA and protein expression were analyzed by the Wilcoxon Median Test. We chose to use a non-parametric test because the transformed cytokine distributions were still slightly skewed, with a number of zero values and high outliers which we did not exclude from the analysis. In addition, because we divided subjects and samples by asthma severity, we had a small sample size for some measures. Finally, in the case of TLR3 and IFN-λ1 mRNAs, which were detected in only 35% and 11% of the samples, data were analyzed as binary variables by Fisher’s exact test .

For our primary analyses, we evaluated the strength of association between mean respiratory tract inflammatory responses and pulmonary function using generalized estimating equations (GEE) with an exchangeable correlation structure using the identity link in the case of continuous outcomes and the log link for binary ones (SAS, Cary, NC). This design allowed us to test the influence of cytokine on lung function with and without virus. GEE was used rather than logistic or Poisson models because of the longitudinal nature of the data and the repeated observations for each child [[4](#_ENREF_4), [5](#_ENREF_5)]. The GEE procedure can be used for small populations [[6](#_ENREF_6)]. All individual samples (virus-negative and virus-positive “surveillance samples,” virus-negative and virus-positive “sick samples”) were included in the analysis. We regressed clinical outcomes on inflammatory response markers, adjusting for the presence of any virus, and including an interaction term between viral presence and inflammatory marker (Model 1). This interaction term allowed us to assess whether the inflammation-outcome relationship was dependent on the presence of virus. If there was no dependence on viral infection (interaction term p≥0.05), the interaction term was not included in the model used for interpretation (Model 2). We adjusted for covariates including age, gender, ethnicity/race, smoker in the home, caregiver educational attainment, self-reported atopy, caregiver depressive symptom score, season and whether the sample was from a surveillance or sick period. Family income, baseline asthma severity, medication use and proximity to high-traffic highways were evaluated but not included in final models as they were not significant predictors or were collinear with other covariates in the model. In a secondary analysis, linear plots of lung function and cytokine level when virus was either absent or present were generated. We did not use a specific adjustment for multiple comparisons, instead, we report all results and highlight the pattern of significant relationships as a valid representation of natural phenomena [[7](#_ENREF_7)]. With this approach, it is recognized that any individual model may spuriously achieve statistically significance (Type I error), but that it is extremely unlikely that multiple models would spuriously show significant relationships of the same magnitude and direction. Thus, omitting corrections for multiple comparisons and identifying consistency of findings across multiple statistical models is seen as a way to avoid Type II error [[8](#_ENREF_8)].

**References**

1. Lewis TC, Robins TG, Dvonch JT, Keeler GJ, Yip FY, Mentz GB, Lin X, Parker EA, Israel BA, Gonzalez L, Hill Y: **Air pollution-associated changes in lung function among asthmatic children in Detroit.** *Environ Health Perspect* 2005, **113:**1068-1075.

2. Hankinson JL, Odencrantz JR, Fedan KB: **Spirometric Reference Values from a Sample of the General U.S. Population.** *Am J Respir Crit Care Med* 1999, **159:**179-187.

3. Mortimer KM, Fallot A, Balmes JR, Tager IB: **Evaluating the use of a portable spirometer in a study of pediatric asthma.** *Chest* 2003, **123:**1899-1907.

4. Liang K-Y, Zeger SL: **Longitudinal data analysis using generalized linear models.** *Biometrika* 1986, **73:**13-22.

5. Ballinger GA: **Using generalized estimating equations for longitudinal data analysis.** *Organ Res Methods* 2004, **7:**127-150.

6. Ma Y, Mazumdar M, Memtsoudis SG: **Beyond repeated-measures analysis of variance: Advanced statistical methods for the analysis of longitudinal data in anesthesia research.** *Reg Anesth Pain Med* 2012, **37:**99-105.

7. Rothman KJ: **No adjustments are needed for multiple comparisons.** *Epidemiology* 1990, **1:**43-46.

8. Feise RJ: **Do multiple outcome measures require p-value adjustment?** *BMC Medical Res Method* 2002, **2:**8-8.

**Figure S1.**

Effect of viral infection on the relationships between log-transformed nasal lavage biomarker levels and percent predicted FVC. In the absence of virus, we found negative associations between biomarker level and FVC (unadjusted 95% confidence intervals are shown in light grey, solid lines indicate a statistically significant association between cytokine and FVC by GEE; dashed lines indicate no statistically significant association). However, in the presence of virus, increasing levels of biomarker had a positive effect on FVC (unadjusted 95% confidence intervals are shown in dark grey; solid lines indicate a statistically significant association).

**Figure S2.** Effect of viral infection on the relationships between log-transformed nasal lavage biomarker levels and percent predicted FEV_1_. In the absence of virus, we found negative associations between biomarker level and FEV_1_ (95% confidence intervals are shown in light grey, with solid lines indicating a significant association between cytokine and FEV_1_ by GEE; dashed lines indicate no statistically significant association). In the presence of virus, increasing levels of biomarker had a positive effect on FEV_1_ (95% confidence intervals are shown in dark grey; solid lines indicate a statistically significant association).

**Table S1.** Classification of asthma and asthma severity based on symptom frequency and reported inhaled steroid use. Relevant questions from the screening questionnaire are shown.

I. A child will be considered to have **probable asthma** (of any severity) if any of the following are true:

1. three or more of the six non-exercise related symptoms (i.e., questions 3, 4, 5, 6, 9, and 10) were reported (at any level of frequency greater than "never")

Q3. In the past 12 months, how often, on average has your child seemed congested in the chest or coughed up phlegm (mucus) when he/she did not have a cold or the flu?

Q4. In the past 12 months, has your child had wheezing or whistling in the chest when he/she had a cold or the flu?

Q5. In the past 12 months, how often, on average has your child had wheezing or whistling in the chest when he/she did not have a cold or the flu?

Q6. In the past 12 months, has your child’s wheezing or whistling in the chest ever been severe enough to limit your child’s speech to only one or two words at a time between breaths?

Q9. In the past 12 months, how often, on average did your child wake up from sleep due to wheezing, dry cough, tightness of the chest, or shortness of breath?

Q10. In the past 12 months, how often, how many days (or part of days) of school has your child missed because of wheezing or asthma?

1. either exercise symptom (i.e., questions 7 and 8) was reported with a frequency of three times or more in the past year.

Q7. In the past 12 months, how often, on average has your sounded wheezy during or after exercise, running, or playing hard?

Q8. In the past 12 months, how often, on average has your child coughed during or after exercise, running, or playing hard?

1. there is a diagnosis of asthma (i.e., yes on question 13) with any symptoms (questions 3 through 10) or doctor-prescribed medication use (i.e., yes on questions 14 and 15)

Q13. Has any doctor, nurse, or other health professional ever said that your child has asthma, reactive airway disease, asthmatic bronchitis or wheezy bronchitis?

Q14. In the past 12 months, has your child taken any medications, inhalers (puffers), or nebulizers (breathing treatments) prescribed by a doctor for any of the conditions just mentioned?

Q15. Does your child take any doctor-prescribed medications for a breathing problem every day, even when he/she is not having trouble breathing?

II. A child will be considered to have **probable moderate to severe asthma** if, first, the child meets the diagnostic criteria for asthma above, and, second, any of the following are true:

1. any daytime symptom (i.e., questions 3 through 9) is reported as being present "every day"
2. sleep disturbance (question 10) is reported "more than *two times* per week" or "most nights"
3. daily use of doctor-prescribed medication use (i.e., yes on questions 14 and 15)

III. A child will be considered to have **probable mild persistent asthma** [of any severity] if, first, the child meets the diagnostic criteria for asthma above, second, the criteria for probable or known moderate to severe asthma are **not** met, and, thirdly, any of the following are true:

1. one or more daytime symptoms are reported as being present "more than 2 times per week"
2. sleep disturbance reported is reported “more than *one time* per month”
3. daily use of doctor-prescribed medication use (i.e., yes on questions 14 and 15)

IV. A child will be considered to have **probable mild intermittent asthma** if, first, the child meets the diagnostic criteria for asthma above, and, second, neither the criteria for probable or known moderate to severe asthma nor the criteria for probable or known mild persistent asthma are met.

**Table S2.** Respiratory symptom score. Total score was the sum of all reported component symptoms.

Mild Moderate Severe

Fever: (1)

Cough: (1) (2) (3)

Runny nose: (1) (2)

Stuffy nose: (1) (2)

Sore throat: (1)

Duration of illness >4 days (1)

Wheezing: (5)

Difficulty Breathing: (5)

Breathing fast: (5)

NOT going to school OR

NOT doing usual activities: (5)

**Table S3.** Primers and probes employed for qPCR.

CXCL8 Forward: TCTGCAGCTCTGTGTGAAGGTGCAGTT

Reverse: AACCCTCTGCACCCAGTTTTCCT

CXCL10 Forward: TTGTCCACGTGTTGAGATCAT

Reverse: TTAGACCTTTCCTTGCTAACTGC

IFN-λ1 Forward: GACTTTGGTGCTAGGCTTGG

Reverse: GGAAGACAGGAGAGCTGCAA

TLR3 Forward: AACGAGACCCATACCAACATCC

Reverse: TGGGAACCCATGATAGTGAGG

MDA-5 Forward: ATGGTCTCGTCACCAATGAA

Reverse: GGACGTAGGTGCTCTCATCA

RIG-I Forward: CCAGATGCCAGACAAAGATG

Reverse: TTCGTGCATGCTCACTGATA

IRF7 Forward: GCGACAGGAGCCCTTACCT

Reverse: CCAAGGCCATTGCTCCTTCT

GAPDH Forward: CGACCACTTTGTCAAGCTCA

Reverse: AGGGGTCTACATGGCAACTG

**Table S4.** Lower limits of detection of muliplex immune assays.

CXCL8 1.2 pg/mL

CXCL10 0.3 pg/mL

IL-4 1.5 pg/mL

IL-13 0.1 pg/mL

sICAM-1 2.72 pg/mL

CCL2 0.6 pg/mL

CCL4 4.7 pg/mL

CCL5 0.2 pg/mL

CCL20 2.0 pg/mL

CCL24 0.34 pg/mL

**Table S5.** Associations of nasal biomarker and percent predicted FVC in the absence of presence of virus. Model 1 includes an interaction term between viral presence and symptom score. If there was no dependence on viral infection, this factor was not included (Model 2).

TLR3 and IFN-λ1 were modeled as present/absent, while other cytokines were modeled as continuous variables on the natural log scale. Significant estimates in the interpreted model are denoted by bold font (p≤0.05).

*Model 1 Model 2*

*Parameter Estimate 95%CI ProbZ Estimate 95%CI ProbZ*

*mRNA* CXCL8 1.20 (-0.3,2.7) 0.12 1.15 (-0.4,2.7) 0.15

virus -1.51 (-8.8,5.8) 0.68 -1.89 (-4.9,1.1) 0.22

virus x CXCL8 -0.22 (-3.8,3.3) 0.91

CXCL10 **-19.06 (-37.4,-0.7) 0.04** not applicable

virus -2.55 (-5.9,0.8) 0.13

virus x CXCL10 **21.91 (2.7,41.1) 0.03**

IRF7 -4.22 (-9.2,0.8) 0.10 -4.18 (-9.2,0.8) 0.10

virus -1.99 (-6,2) 0.32 -1.96 (-5,1) 0.20

virus x IRF7 0.29 (-17.6,18.2) 0.97

RIG-I 2.88 (-7.9,13.7) 0.60 1.99 (-7.8,11.8) 0.69

virus -1.67 (-5.4,2.1) 0.38 -1.88 (-4.9,1.1) 0.22

virus x RIG-I -5.66 (-40.8,29.5) 0.75

MDA5 -19.18 (-38.1,-0.3) 0.05 -13.93 (-33.7,5.9) 0.17

virus -2.81 (-6.8,1.1) 0.16 -1.72 (-4.8,1.3) 0.27

virus x MDA5 26.68 (-9.1,62.4) 0.14

TLR3 -3.97 (-8.26,0.31) 0.07 not applicable

virus **-5.62 (-9.32,-1.91) <0.01**

virus x TLR3 **10.38 (3.60,17.17) <0.01**

IFNλ1 -4.09 (-12.72,4.54) 0.35 -2.03 (-7.12,3.06) 0.43

virus -2.10 (-5.71,1.51) 0.25 -1.60 (-477,1.57) 0.32

virus x IFNλ1 4.06 (-6.41,14.54) 0.45

protein CXCL8 -1.00 (-2.25,0.24) 0.11 -0.44 (-1.89,1) 0.55

virus -10.81 (-23.67,2.04) 0.10 -2.66 (-6.23,0.91) 0.14

virus x CXCL8 1.56 (-0.73,3.85) 0.18

CXCL10 -3.02 (-5.4,-0.65) 0.01 **-2.24 (-3.93,-0.55) 0.01**

virus -14.62 (-38.03,8.78) 0.22 -1.55 (-5.09,1.99) 0.39

virus x CXCL10 2.11 (-1.52,5.74) 0.26

IL-4 **-1.54 (-2.68,-0.39) 0.01** -1.03 (-2.02,-0.04) 0.04

virus -9.96 (-20.54,0.62) 0.07 -1.86 (-5.16,1.44) 0.27

virus x IL-4 **2.50 (-0.08,5.09) 0.05**

(continued)

IL-13 **-1.36 (-2.33,-0.39) 0.01** not applicable

virus **-6.89 (-13.04,-0.74) 0.03**

virus x IL-13 **1.80 (0.18,3.41) 0.03**

ICAM-1 -1.33 (-2.44,-0.22) 0.02 *-*0.96 (-2,0.08) 0.07

virus -12.15 (-30.65,6.35) 0.20 -1.62 (-5.07,1.83) 0.36

virus x ICAM-1 1.78 (-1.01,4.56) 0.21

CCL2 -0.78 (-1.76,0.19) 0.11 -0.57 (-1.53,0.39) 0.25

virus -7.00 (-21.06,7.06) 0.33 -2.36 (-5.89,1.16) 0.19

virus x CCL2 1.12 (-1.74,3.99) 0.44

CCL4 -0.53 (-1,-0.05) 0.03 -0.23 (-0.7,0.23) 0.32

virus -8.65 (-17.68,0.38) 0.06 -3.10 (-7,0.8) 0.12

virus x CCL4 1.01 (-0.15,2.18) 0.09

CCL5 **-1.36 (-2.45,-0.27) 0.01** not applicable

virus **-7.57 (-14.55,-0.59) 0.03**

virus x CCL5 **2.47 (0.25,4.7) 0.03**

CCL20 **-1.57 (-2.53,-0.6) 0.00** not applicable

virus **-15.63 (-28.6,-2.67) 0.02**

virus x CCL20 **2.42 (0.51,4.34) 0.01**

CCL24 **-1.25 (-1.91,-0.59) 0.00** not applicable

virus **-10.50 (-19.91,-1.08) 0.03**

virus x CCL24 **2.11 (0.28,3.94) 0.02**

**Table S6.** Associations of nasal biomarker and percent predicted FEV_1_ in the absence of presence of virus. Model 1 includes an interaction term between viral presence and symptom score. If there was no dependence on viral infection, this factor was not included (Model 2).

TLR3 and IFN-λ1 were modeled as present/absent, while other cytokines were modeled as continuous variables on the natural log scale. Significant estimates in the interpreted model are denoted by bold font (p≤0.05).

*Model 1 Model 2*

*Parameter Estimate 95%CI ProbZ Estimate 95%CI ProbZ*

mRNA CXCL8 1.30 (-1.22,3.83) 0.31 0.72 (-1.49,2.94) 0.52

virus 1.62 (-6.85,10.08) 0.71 -1.88 (-4.99,1.22) 0.23

virus x CXCL8 -1.93 (-6.45,2.58) 0.40

CXCL10 **-32.82 (-49.62,-16.02) <0.01** -1.85 (-9.91,6.20) 0.65

virus -2.86 (-6.29,0.57) 0.10 -1.67 (-5.01,1.67) 0.33

virus x CXCL10 **36.12 (17.94,54.30) <0.01**

IRF7 -2.10 (-6.10,1.90) 0.30 -2.36 (-6.36,1.63) 0.25

virus -1.66 (-5.57,2.25) 0.41 -1.89 (-4.99,1.21) 0.23

virus x IRF7 -2.21 (-18.40,13.98) 0.79

RIG-I -16.38 (-24.34,-8.42) <0.01 **-12.48 (-21.50,-3.47) 0.01**

virus -2.61 (-6.27,1.06) 0.16 -1.72 (-4.80,1.35) 0.27

virus x RIG-I 22.52 (-8.84,53.88) 0.16

MDA5 -16.70 (-28.70,-4.71) 0.01 **-12.87 (-23.73,-2.01) 0.02**

virus -2.79 (-6.68,1.09) 0.16 -1.67 (-4.74,1.40) 0.29

virus x MDA5 24.93 (-4.37,54.23) 0.10

TLR3 -1.84 (-5.89,2.22) 0.37 1.24 (-2.22,4.69) 0.48

virus **-5.67 (-9.20,-2.14) <0.01** -1.98 (-5.03,1.06) 0.20

virus x TLR3 **9.97 (2.56,17.37) 0.01**

IFN-λ1 -4.81 (-12.97,3.34) 0.25 -2.53 (-7.85,2.79) 0.35

virus -2.12 (-5.70,1.46) 0.25 -1.53 (-4.81,1.76) 0.36

virus x IFN-λ1 4.78 (-5.07,14.62) 0.34

protein CXCL8 -0.82 (-1.83,0.19) 0.11 -0.53 (-1.92,0.87) 0.46

virus -7.00 (-20.81,6.81) 0.32 -2.92 (-6.57,0.73) 0.12

virus x CXCL8 0.78 (-1.77,3.33) 0.55

CXCL10 -2.15 (-4.44,0.14) 0.07 **-1.86 (-3.52,-0.19) 0.03**

virus -6.71 (-31.78,18.37) 0.60 -1.67 (-5.38,2.05) 0.38

virus x CXCL10 0.81 (-3.12,4.75) 0.69

IL-4 -0.88 (-2.12,0.36) 0.16 -0.27 (-1.2,0.65) 0.57

virus **-11.58 (-21.43,-1.73) 0.02** -2.35 (-5.86,1.16) 0.19

virus x IL-4 **2.87 (-0.04,5.77) 0.05**

(continued)

IL-13 -0.82 (-1.89,0.25) 0.13 -0.42 (-1.33,0.48) 0.36

virus -6.69 (-13.11,-0.27) 0.04 -3.23 (-7.04,0.58) 0.10

virus x IL-13 1.51 (-0.59,3.61) 0.16

ICAM-1 -0.30 (-1.19,0.58) 0.50 0.06 (-0.73,0.85) 0.88

virus -11.93 (-27.13,3.26) 0.12 -2.41 (-5.89,1.06) 0.17

virus x ICAM-1 1.61 (-0.86,4.08) 0.20

CCL2 -0.05 (-0.88,0.77) 0.90 0.24 (-0.5,0.99) 0.52

virus -8.48 (-17.38,0.42) 0.06 -2.79 (-6.33,0.75) 0.12

virus x CCL2 1.38 (-0.72,3.48) 0.20

CCL4 -0.25 (-0.74,0.23) 0.31 0.04 (-0.44,0.52) 0.88

virus -8.39 (-15.58,-1.2) 0.02 -3.12 (-6.84,0.59) 0.10

virus x CCL4 0.97 (-0.17,2.1) 0.09

CCL5 -0.58 (-1.82,0.67) 0.37 0.08 (-0.88,1.04) 0.87

virus -7.94 (-14.54,-1.35) 0.02 -2.70 (-6.26,0.86) 0.14

virus x CCL5 2.45 (-0.11,5.01) 0.06

CCL20 -0.79 (-1.74,0.16) 0.10 -0.34 (-1.17,0.48) 0.41

virus -11.40 (-26.96,4.16) 0.15 -2.37 (-5.58,0.84) 0.15

virus x CCL20 1.55 (-0.98,4.09) 0.23

CCL24 -0.57 (-1.31,0.17) 0.13 -0.13 (-0.81,0.54) 0.70

virus **-9.70 (-17.08,-2.32) 0.01** -2.53 (-6.02,0.96) 0.16

virus x CCL24 **1.80 (0.04,3.56) 0.04**

**Table S7.**  Associations of nasal biomarker and percent predicted FEV_1_/FVC ratio in the absence of presence of virus. Model 1 includes an interaction term between viral presence and symptom score. If there was no dependence on viral infection, this factor was not included (Model 2). TLR3 and IFN-λ1 were modeled as present/absent, while other cytokines were modeled as continuous variables on the natural log scale. Significant estimates in the interpreted model are denoted by bold font (p≤0.05).

*Model 1 Model 2*

*Parameter Estimate 95%CI ProbZ Estimate 95%CI ProbZ*

mRNA CXCL8 0.84 (-0.97,2.65) 0.36 0.23 (0.81,1.82) 0.77

virus 3.60 (-1.11,8.31) 0.13 -0.10 (1.12,2.10) 0.93

virus x CXCL8 -2.08 (-4.54,0.38) 0.10

CXCL10 **-13.44 (-22.42,-4.46) <0.01** -1.85 (2.42,2.89) 0.44

virus -0.36 (-2.74,2.03) 0.77 0.07 (1.17,2.35) 0.95

virus x CXCL10 **13.42 (2.60,24.23) 0.02**

IRF7 1.47 (-3.43,6.37) 0.56 1.05 (2.41,5.77) 0.66

virus 0.30 (-2.60,3.21) 0.84 -0.07 (1.12,2.13) 0.95

virus x IRF7 -3.60 (-21.07,13.86) 0.69

RIG-I -12.42 (-22.51,-2.33) 0.02 **-10.74 (4.99,-0.96) 0.03**

virus -0.42 (-2.89,2.04) 0.74 -0.02 (1.09,2.12) 0.99

virus x RIG-I 10.62 (-15.11,36.34) 0.42

MDA5 -5.59 (-17.67,6.49) 0.36 -6.31 (5.54,4.54) 0.25

virus 0.12 (-2.64,2.89) 0.93 -0.03 (1.10,2.12) 0.98

virus x MDA5 -3.65 (-33.79,26.49) 0.81

TLR3 0.05 (-2.05,2.15) 0.96 0.70 (0.90,2.46) 0.44

virus -0.95 (-3.72,1.82) 0.50 -0.17 (1.10,1.99) 0.88

virus x TLR3 2.11 (-2.33,6.54) 0.35

IFN-λ1 -0.12 (-3.15,2.92) 0.94 -0.20 (1.23,2.22) 0.87

virus -0.05 (-2.58,2.49) 0.97 -0.07 (1.16,2.21) 0.95

virus x IFN-λ1 -0.17 (-5.04,4.69) 0.94

protein CXCL8 -0.32 (-1.18,0.55) 0.47 -0.54 (-1.33,0.25) 0.18

virus 3.04 (-7.24,13.33) 0.56 -0.20 (-2.58,2.17) 0.87

virus x CXCL8 -0.62 (-2.44,1.2) 0.50

CXCL10 0.09 (-1.55,1.72) 0.92 -0.31 (-1.52,0.91) 0.62

virus 6.93 (-9.68,23.54) 0.41 0.29 (-2.28,2.86) 0.82

virus x CXCL10 -1.07 (-3.65,1.51) 0.42

IL-4 0.14 (-0.58,0.85) 0.71 0.27 (-0.46,0.99) 0.47

virus -2.24 (-9.04,4.55) 0.52 -0.17 (-2.6,2.26) 0.89

virus x IL-4 0.64 (-1.34,2.62) 0.53

(continued)

IL-13 0.31 (-0.29,0.9) 0.31 0.22 (-0.38,0.83) 0.47

virus 0.53 (-3.79,4.84) 0.81 -0.21 (-2.76,2.35) 0.87

virus x IL-13 -0.32 (-1.81,1.17) 0.68

ICAM-1 0.56 (-0.22,1.35) 0.16 0.53 (-0.13,1.19) 0.11

virus 0.74 (-13.13,14.6) 0.92 -0.26 (-2.74,2.22) 0.84

virus x ICAM-1 -0.17 (-2.41,2.07) 0.88

CCL2 0.56 (-0.14,1.26) 0.12 0.56 (-0.17,1.29) 0.13

virus -0.52 (-10.48,9.45) 0.92 -0.45 (-2.95,2.05) 0.72

virus x CCL2 0.01 (-2.22,2.25) 0.99

CCL4 0.15 (-0.23,0.52) 0.45 0.16 (-0.14,0.47) 0.29

virus -0.42 (-6.72,5.87) 0.90 -0.08 (-2.66,2.49) 0.95

virus x CCL4 0.06 (-0.93,1.06) 0.90

CCL5 0.50 (-0.16,1.17) 0.14 *0.56 (-0.02,1.13) 0.06*

virus -0.79 (-5.15,3.57) 0.72 -0.37 (-2.81,2.08) 0.77

virus x CCL5 0.20 (-1.55,1.95) 0.82

CCL20 0.43 (-0.36,1.21) 0.29 0.30 (-0.39,1) 0.39

virus 2.20 (-8.18,12.58) 0.68 -0.40 (-2.8,2.01) 0.75

virus x CCL20 -0.45 (-2.14,1.25) 0.61

CCL24 0.41 (-0.13,0.95) 0.14 0.42 (-0.1,0.94) 0.11

virus -0.50 (-6.9,5.9) 0.88 -0.31 (-2.67,2.05) 0.80

virus x CCL24 0.05 (-1.44,1.54) 0.95

**Table S8.** Associations of nasal biomarker and percent predicted FEF_25-75_ in the absence of presence of virus. Model 1 includes an interaction term between viral presence and symptom score. If there was no dependence on viral infection, this factor was not included (Model 2).

TLR3 and IFN-λ1 were modeled as present/absent, while other cytokines were modeled as continuous variables on the natural log scale. Significant estimates in the interpreted model are denoted by bold font (p≤0.05).

*Model 1 Model 2*

*Parameter Estimate 95%CI ProbZ Estimate 95%CI ProbZ*

*mRNA* CXCL8 -2.19 (-5.3,0.9) 0.17 **-2.60 (-5.1,-0.2) 0.04**

virus -1.98 (-13.5,9.6) 0.74 **-4.49 (-8.3,-0.7) 0.02**

virus x CXCL8 -1.40 (-7.1,4.3) 0.63

CXCL10 **-19.74 (-38.7,-0.8) 0.04** 2.67 (-6.3,11.6) 0.56

virus **-5.70 (-10,-1.4) 0.01** -4.85 (-9.1,-0.6) 0.03

virus x CXCL10 **25.95 (4.6,47.3) 0.02**

IRF7 -1.35 (-7.4,4.7) 0.66 -1.23 (-7.2,4.7) 0.68

virus -4.73 (-10.6,1.1) 0.11 **-4.62 (-8.7,-0.5) 0.03**

virus x IRF7 1.04 (-29.9,32) 0.95

RIG-I -13.22 (-28.4,2) 0.09 -11.83 (-28.6,4.9) 0.17

virus -4.84 (-9.5,-0.1) 0.04 **-4.49 (-8.5,-0.5) 0.03**

virus x RIG-I 8.78 (-52,69.6) 0.78

MDA5 -12.92 (-24.7,-1.1) 0.03 -13.10 (-29.2,3) 0.11

virus -4.39 (-9.4,0.7) 0.09 **-4.43 (-8.4,-0.5) 0.03**

virus x MDA5 -0.87 (-66.1,64.3) 0.98

TLR3 -2.29 (-6.6,2.0) 0.30 -0.54 (-4.0,3.0) 0.76

virus -6.68 (-11.6, -1.8) 0.01 **-4.52 (-8.69.-0.5) 0.03**

virus x TLR3 5.68 (-2.4,13.7) 0.17

IFN-λ1 -1.32 (-9.5,6.9) 0.75 3.35 (-3.6,10.3) 0.35

virus -6.28 (-11.8,-0.7) 0.03 **-5.06 (-9.4,-0.7) 0.02**

virus x IFNλ1 9.18 (-6.0,24.3) 0.23

*protein* CXCL8 -1.75 (-3.46,-0.05) 0.04 **-2.17 (-3.51,-0.83) 0.00**

virus 0.80 (-16.75,18.35) 0.93 **-5.59 (-9.97,-1.22) 0.01**

virus x CXCL8 -1.22 (-4.17,1.73) 0.42

CXCL10 -1.59 (-4.24,1.06) 0.24 -1.71 (-3.5,0.09) 0.06

virus -2.46 (-30.71,25.78) 0.86 -4.34 (-9.1,0.42) 0.07

virus x CXCL10 -0.30 (-4.5,3.89) 0.89

IL-4 -0.46 (-1.81,0.89) 0.50 -0.19 (-1.45,1.08) 0.77

virus -10.09 (-22.02,-1.84) 0.01 **-5.87 (-10.62,-1.13) 0.02**

virus x IL-4 1.28 (-2.25,4.82) 0.48

(continued)

IL-13 0.15 (-1.01,1.3) 0.80 0.33 (-0.68,1.33) 0.52

virus -6.88 (-13.04,-0.71) 0.03 **-5.28 (-9.86,-0.69) 0.02**

virus x IL-13 0.68 (-1.54,2.91) 0.55

ICAM-1 -0.50 (-1.89,0.88) 0.48 -0.52 (-1.73,0.7) 0.40

virus -4.15 (-24.25,15.95) 0.69 **-4.53 (-8.96,-0.1) 0.05**

virus x ICAM-1 -0.06 (-3.34,3.22) 0.97

CCL2 0.16 (-1.06,1.39) 0.79 -0.01 (-1.27,1.25) 0.99

virus -1.72 (-19.34,15.9) 0.85 **-5.68 (-10.45,-0.9) 0.02**

virus x CCL2 -0.94 (-5.1,3.21) 0.66

CCL4 -0.16 (-0.9,0.58) 0.67 0.01 (-0.59,0.61) 0.97

virus -9.36 (-21.55,2.82) 0.13 **-5.65 (-10.35,-0.95) 0.02**

virus x CCL4 0.66 (-1.09,2.41) 0.46

CCL5 0.38 (-1.08,1.84) 0.61 0.52 (-0.67,1.7) 0.39

virus -7.05 (-14.01,-0.08) 0.05 **-5.90 (-10.55,-1.26) 0.01**

virus x CCL5 0.52 (-2.6,3.64) 0.74

CCL20 -0.36 (-1.67,0.94) 0.58 -0.46 (-1.38,0.47) 0.33

virus -3.77 (-20.26,12.73) 0.65 **-5.63 (-9.98,-1.29) 0.01**

virus x CCL20 -0.32 (-3.04,2.41) 0.82

CCL24 -0.31 (-1.23,0.61) 0.51 -0.25 (-1,0.5) 0.51

virus -6.95 (-16.92,3.03) 0.17 **-5.94 (-10.59,-1.29) 0.01**

virus x CCL24 0.25 (-2.25,2.74) 0.85

**Table S9.** Associations of nasal biomarker and percent predicted PEF in the absence of presence of virus. Model 1 includes an interaction term between viral presence and symptom score. If there was no dependence on viral infection, this factor was not included (Model 2).

TLR3 and IFN-λ1 were modeled as present/absent, while other cytokines were modeled as continuous variables on the natural log scale. Significant estimates in the interpreted model are denoted by bold font (p≤0.05).

*Model 1 Model 2*

*Parameter Estimate 95%CI ProbZ Estimate 95%CI ProbZ*

*mRNA* CXCL8 1.52 (-2.09,5.14) 0.41 0.64 (-2.67,3.95) 0.71

virus 2.67 (-7.45,12.78) 0.61 -2.67 (-6.56,1.23) 0.18

virus x CXCL8 -2.95 (-8.66,2.77) 0.31

CXCL10 **-39.23 (-57.34,-21.13) <0.01** -4.10 (-12.21, 4.01) 0.32

virus -3.63 (-8.23,0.97) 0.12 -2.27 (-6.45,1.91) 0.29

virus x CXCL10 **40.99 (20.14,61.85) <0.01**

IRF7 -2.15 (-8.22,3.92) 0.49 -3.04 (-9.22,3.14) 0.34

virus -1.94 (-6.91,3.03) 0.44 -2.70 (-6.64,1.25) 0.18

virus x IRF7 -7.38 (-26.32,11.57) 0.45

RIG-I **-31.02 (-39.42,-22.62) <0.01** -25.77 (-35.48,-16.06) <0.01

virus -3.59 (-8.01,0.83) 0.11 -2.40 (-6.34,1.53) 0.23

virus x RIG-I **30.37 (-0.42,61.15) 0.05**

MDA5 -21.33 (-38.55,-4.11) 0.02 **-17.05 (-31.06,-3.04) 0.02**

virus -3.67 (-8.56,1.22) 0.14 -2.41 (-6.32,1.49) 0.23

virus x MDA5 27.76 (-10.42,65.93) 0.15

TLR3 0.48 (-5.05,6.01) 0.87 3.80 (-0.88,8.49) 0.11

virus **-7.05 (-11.86,-2.24) <0.01** -3.09 (-6.85,0.67) 0.11

virus x TLR3 **10.72 (1.13,20.31) 0.03**

IFN-λ1 -2.14 (-12.07,7.79) 0.67 -2.08 (-8.65,4.49) 0.53

virus -2.39 (-6.84,2.07) 0.29 -2.37 (-6.51,1.77) 0.26

virus x IFNλ1 0.12 (-12.33,12.56) 0.99

protein CXCL8 -0.97 (-2.66,0.72) 0.26 -0.86 (-2.69,0.97) 0.36

virus -5.62 (-23.02,11.79) 0.53 -4.07 (-8.67,0.53) 0.08

virus x CXCL8 0.30 (-2.97,3.57) 0.86

CXCL10 -0.14 (-2.69,2.42) 0.92 -0.53 (-2.31,1.25) 0.56

virus 3.45 (-28.01,34.92) 0.83 -3.09 (-7.79,1.6) 0.20

virus x CXCL10 -1.06 (-5.87,3.75) 0.67

IL-4 -0.23 (-1.63,1.16) 0.74 0.30 (-0.86,1.45) 0.61

virus -11.78 (-23.32,-0.24) 0.05 -3.90 (-8.36,0.55) 0.09

virus x IL-4 2.44 (-0.69,5.58) 0.13

(continued)

IL-13 -0.20 (-1.38,0.98) 0.74 -0.12 (-1.07,0.84) 0.81

virus -4.90 (-13.27,3.46) 0.25 -4.15 (-9.11,0.8) 0.10

virus x IL-13 0.33 (-2.22,2.87) 0.80

ICAM-1 0.18 (-1.07,1.43) 0.78 0.33 (-0.74,1.39) 0.55

virus -7.75 (-26.66,11.17) 0.42 -3.86 (-8.43,0.71) 0.10

virus x ICAM-1 0.66 (-2.34,3.65) 0.67

CCL2 0.29 (-1.1,1.68) 0.68 0.57 (-0.64,1.79) 0.35

virus -9.39 (-22.06,3.29) 0.15 -3.92 (-8.35,0.51) 0.08

virus x CCL2 1.33 (-1.45,4.1) 0.35

CCL4 -0.23 (-0.89,0.43) 0.49 0.01 (-0.67,0.69) 0.98

virus -8.35 (-18.04,1.33) 0.09 -4.00 (-8.83,0.84) 0.11

virus x CCL4 0.80 (-0.66,2.26) 0.28

CCL5 0.29 (-1.17,1.75) 0.69 0.85 (-0.37,2.07) 0.17

virus -8.71 (-17.76,0.34) 0.06 -4.32 (-9.03,0.4) 0.07

virus x CCL5 2.06 (-0.93,5.04) 0.18

CCL20 -0.94 (-2.42,0.54) 0.21 -0.59 (-1.85,0.67) 0.36

virus -10.91 (-29.36,7.55) 0.25 -3.84 (-8.03,0.34) 0.07

virus x CCL20 1.21 (-1.71,4.14) 0.42

CCL24 -0.45 (-1.52,0.63) 0.42 -0.07 (-1.05,0.9) 0.88

virus -9.94 (-19.53,-0.36) 0.04 -3.82 (-8.36,0.72) 0.10

virus x CCL24 1.54 (-0.58,3.66) 0.15
