# Supplementary figures and images for: Influence of viral infection on the relationships between airway cytokines and lung function in asthmatic children
Source: Respir Res. 2018 Nov 21;19:228. doi: 10.1186/s12931-018-0922-9 (PMC6249926; doi:10.1186/s12931-018-0922-9)

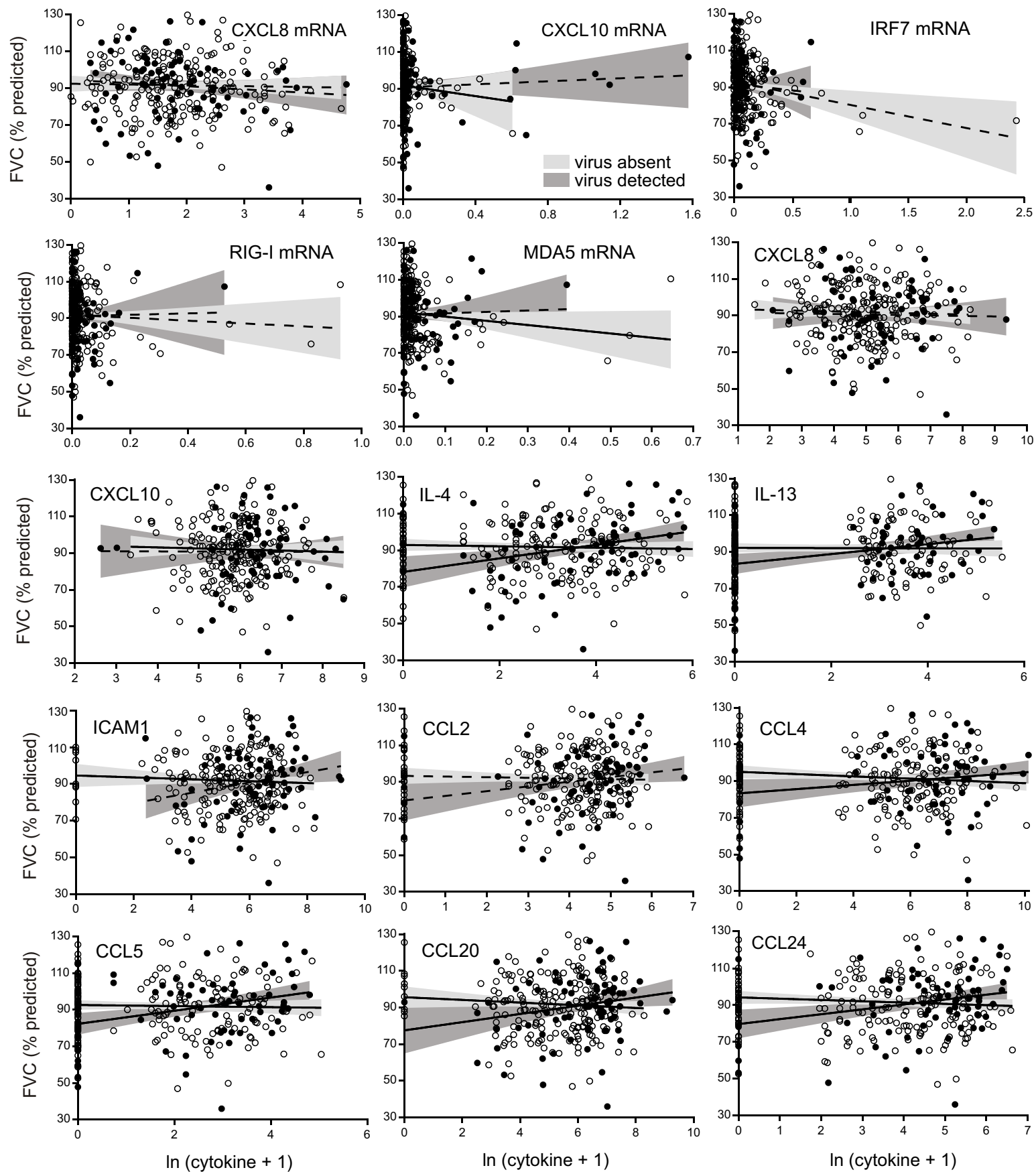

Figure S1

Supplement: Supplementary file 2 — Figure S1. (PDF 13647 kb) [file 12931_2018_922_MOESM2_ESM.pdf]

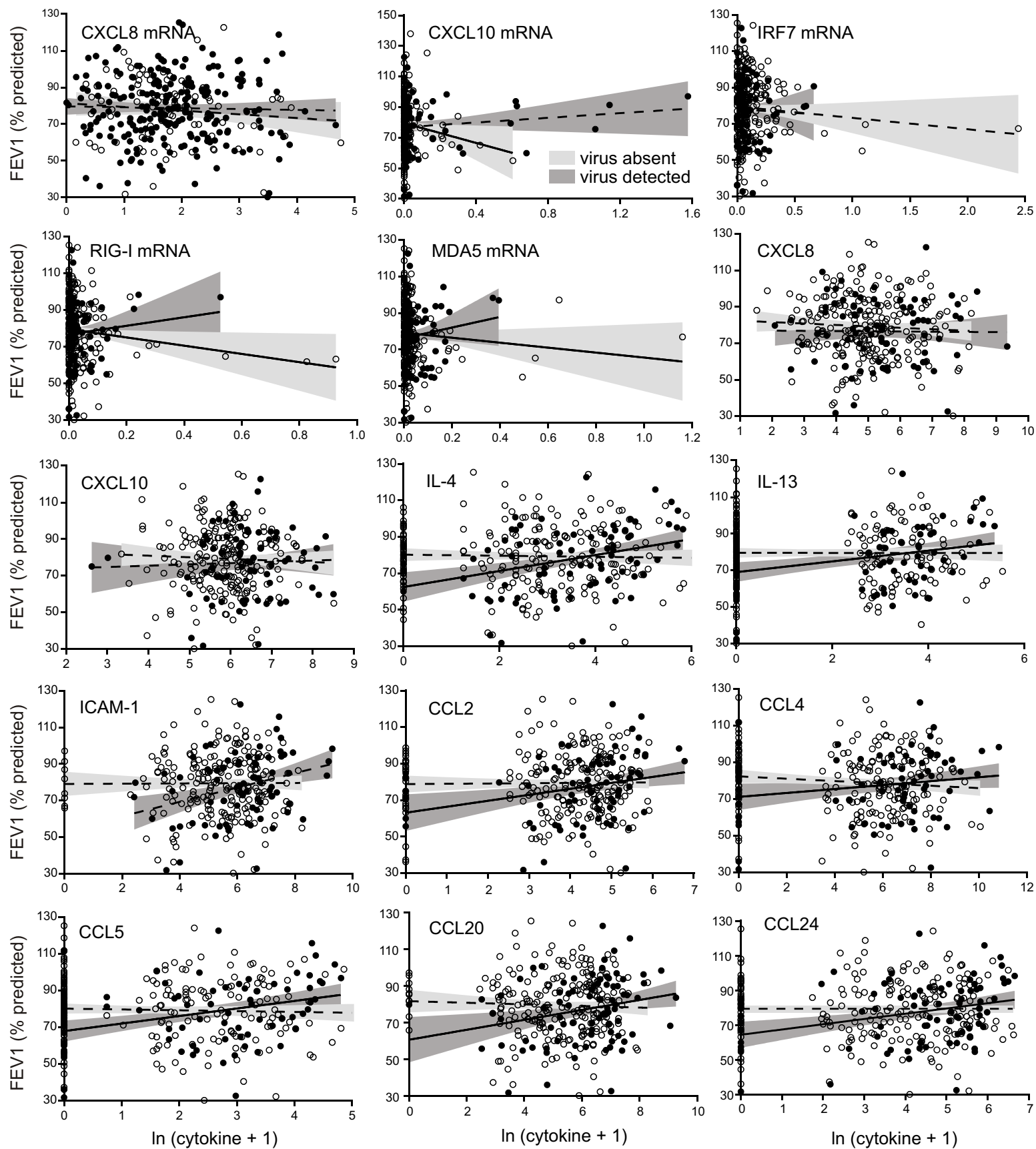

Figure S2

Supplement: Supplementary file 3 — Figure S2. (PDF 14140 kb) [file 12931_2018_922_MOESM3_ESM.pdf]
